# Impact of using data from electronic protocols in nursing performance management: A qualitative interview study

**DOI:** 10.1111/jonm.12858

**Published:** 2019-10-28

**Authors:** Joanna Hope, Peter Griffiths, Paul E. Schmidt, Alejandra Recio‐Saucedo, Gary B. Smith

**Affiliations:** ^1^ School of Health Sciences National Institute for Health Research (NIHR) Collaboration for Applied Health Research and Care (CLAHRC) University of Southampton Wessex, Southampton UK; ^2^ School of Health Sciences University of Southampton Southampton UK; ^3^ Portsmouth Hospitals NHS Trust Medical Assessment Unit Queen Alexandra Hospital Portsmouth UK; ^4^ School of Health Sciences University of Southampton Southampton UK; ^5^ Centre of Postgraduate Medical Research & Education (CoPMRE) Faculty of Health and Social Sciences Bournemouth University Bournemouth Dorset UK

**Keywords:** early warning score, information management and technology, nursing quality, patient safety, performance management

## Abstract

**Aim:**

To explore the impact of using electronic data in performance management to improve nursing compliance with a protocol.

**Background:**

Electronic data are increasingly used to monitor protocol compliance but little is known about the impact on nurses’ practice in hospital wards.

**Method:**

Seventeen acute hospital nursing staff participated in semi‐structured interviews about compliance with an early warning score (EWS) protocol delivered by a bedside electronic handheld device.

**Results:**

Before electronic EWS data was used to monitor compliance, staff combined protocol‐led actions with clinical judgement. However, some observations were missed to reduce noise and disruption at night. After compliance monitoring was introduced, observations were sometimes covertly omitted using a loophole. Interviewees described a loss of autonomy but acknowledged the EWS system sometimes flagged unexpected patient deterioration.

**Conclusions:**

Introducing automated electronic systems to support nursing tasks can decrease nursing burden but remove the ability to record legitimate reasons for missing observations. This can result in covert resistance that could reduce patient safety.

**Implications for nursing management:**

Providing the ability to log legitimate reasons for missing observations would allow nurses to balance professional judgement with the use of electronic data in performance management of protocol compliance.

## 1 INTRODUCTION

Omissions in nursing care are increasingly recommended as nursing quality measures (Griffiths et al., 2018; VanFosson, Jones, & Yoder, 2016). In part, this reflects a move away from relying solely on professional judgement (Allen, 2009) towards auditable systems and procedures (Power, 1997), particularly in nursing care (Pinder, Petchey, Shaw, & Carter, 2006). When protocols are operationalized through electronic devices the resulting data create new ways of measuring nursing omissions. For instance, the National Health Service (NHS), the national, free‐at‐the‐point‐of‐entry healthcare system, provides access to advice from healthcare professionals via its 111 telephone call service. Time‐stamped electronic data collected during these calls are used to compare nurse response times and numbers of enquiries resolved within the time required by their protocol (Pope et al., 2017; Prichard, Turnbull, Halford, & Pope, 2014; Ruston, 2006). Previous research has shown that nurses can resist electronically based protocol implementation in overt and covert ways (Pope et al., 2017; Prichard et al., 2014; Ruston, 2006; Timmons, 2003), thereby undermining the aims of the protocol (Timmermans & Berg, 2003). The research to date has focused on NHS 111 (Pope et al., 2017; Prichard et al., 2014; Ruston, 2006) electronic patient records (e.g. Timmons, 2003), or interactive whiteboards (Allen, 2014), which are not time critical. It is therefore important to understand the impact of using electronic data in time‐dependent protocols for performance management in a ward with multiple task demands.

In this research, we explore the use of early warning score (EWS) protocols embedded within bedside electronic handheld devices. Taking and interpreting vital signs are a fundamental aspect of nursing care (Kitson, Conroy, Wengstrom, Profetto‐McGrath, & Robertson‐Malt, 2010). Early warning scores were introduced as a standardized way to measure patient deterioration (Morgan, Williams, & Wright, 1997), weighting physiological readings based on degree of deviation from agreed normal ranges to create a score capturing overall severity of patient illness. Examples include the National EWS (NEWS; Royal College of Physicians, 2012), NEWS2 (Royal College of Physicians, 2017) and the Modified EWS (MEWS; Morgan et al., 1997). The associated escalation actions at different levels of EWS value vary according to the locally agreed protocol. Reduced intervals between observations are recommended when EWS values increase (Royal College of Physicians, 2012, 2017) but the optimum measurement frequency and combination of vital signs is currently unknown (Smith, Recio‐Saucedo, & Griffiths, 2017). There has been a high uptake of EWS protocols with 99% (530/538) of UK hospitals using them to monitor deteriorating patients, with 97.9% using linked escalation actions (National Confidential Enquiry into Patient Outcome and Death [NCEPOD], 2015).

One reason the use of electronic EWS protocols has increased is because of Francis report recommendations that automated vital signs taking could improve patient safety (Francis, 2013, p. 1599) and research highlighting inaccuracies in paper‐based EWS scores (Niegsch, Fabritius, & Anhøj, 2013; Odell, 2015). The introduction of electronic EWS systems has been associated with decreased mortality in hospitals (Schmidt et al., 2015) and significant improvement in clinical responses (Credland, Dyson, & Johnson, 2018; Jones et al., 2011). Yet non‐compliance persists at night and for patients with the highest EWS values (Griffiths et al., 2018; Hands et al., 2013; Jones et al., 2011). While some non‐compliance is associated with low staffing levels, most is not (Griffiths et al., 2018).

This paper explores how electronic data were used to performance manage ward‐level nursing compliance with a EWS protocol. A 'technologies of practice' perspective is employed, which emphasizes the importance of understanding the impact of technology on a social setting and vice versa (Timmermans & Berg, 2003).

Aim: To explore the impact of using electronic data in performance management to improve nursing compliance with a care protocol.

## 2 METHODOLOGY

### 2.1 Background

**Figure 1:**
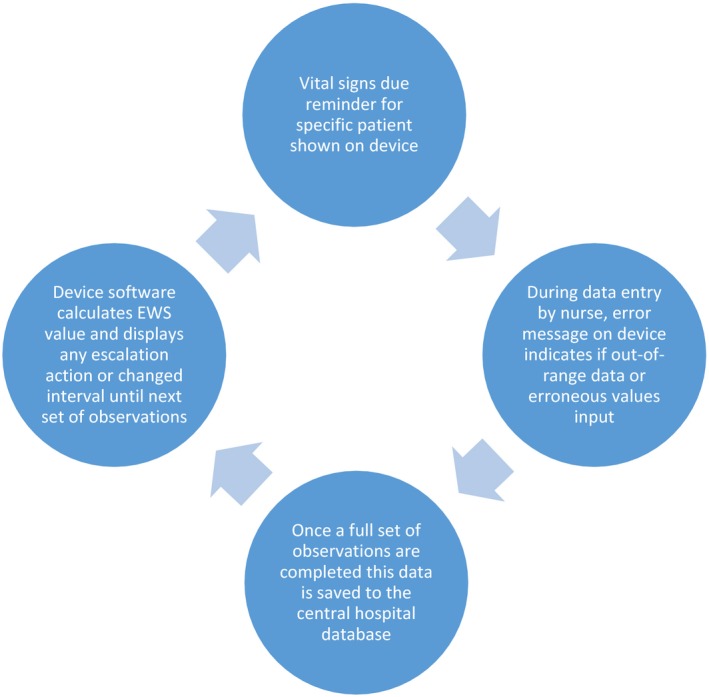
How devices link to the electronic early warning score protocol [Colour figure can be viewed at https://www.wileyonlinelibrary.com]

The data in this study derives from a wider study exploring why diurnal variation in vital signs observations persisted in the study hospital following the introduction of a EWS protocol embedded within bedside handheld devices. The study hospital uses commercially available, small handheld electronic devices in all adult inpatient wards except maternity and high care areas. The embedded software requires nurse input of a full vital signs set required by the EWS algorithm. Once a full set is entered, a timestamp is assigned to the data, which is saved to the hospital's central database. The EWS value is automatically calculated and any required actions are displayed on the device screen. The interval of observation varies by the EWS value, with the highest value requiring observation sets every 30 min and the lowest every 12 hr. For patients being monitored continuously, vital signs should be entered manually into the device. Figure 1 summarizes this process. The time due to the observation is shown in rounded hours. When <25% of the interval is left, a white clock is displayed on the device screens. When the due time is reached, an amber clock icon appears. This turns red if an observation is overdue by 30% past the scheduled interval. A summary for all patients is visible on linked software on ward computers and tablets.

During the study period, the hospital started using the centralized, time‐stamped EWS data to assess ward compliance with scheduled EWS observations. The target was to take vital sign observations within 133% of the scheduled time, 80% of the time (e.g. when hourly observations were scheduled, they were considered late if 80 or more minutes had elapsed since last observation set was recorded). Wards were judged against each other and ward leaders tasked with improving or maintaining compliance levels.

## 3 METHODS

The project received University ethical approval on 30/03/2015 with governance approval gained from the hospital's Research and Development Office on 15/06/2015. This was a qualitative interpretative study using semi‐structured interviews. A qualitative approach was chosen as it is the method of choice for exploring implementation of technical solutions in health care (Berg, 1997). Members of staff in a general hospital in the South of England were recruited through a survey exploring night‐time compliance with the protocol. Seventy survey respondents indicated their interest in participating; forty‐eight were eligible and provided accurate contact details. For inclusion, staff were required to have worked night shifts immediately prior to the survey launch. Attempts were made to build a “deviant case sample” (Patton, 2015), recruiting wards with the highest and lowest night‐time protocol compliance. However, this had to be expanded to all eligible interested staff to ensure recruitment was high enough to reach data saturation. Seventeen staff members participated in interviews in June 2016. This sample resembled a maximum variation sample in terms of ward compliance level, years of experience and speciality (see Table 1). Ward compliance was measured by using administrative data from the hospital to stratify wards into quartiles reflecting percentage of scheduled vital sign observations taken on time at night according to the EWS protocol (see Table 1). Wards represented had six to 65 beds (including trolleys and chairs). The hospital runs at high occupancy (90%–95%). The average Registered Nurse Care Hours Per Patient Day varied between 3.8 in surgical wards to 7.4 in the Medical Assessment Unit (MAU), and average Health Care Assistant hours per patient day from 2.8 in medical wards to 3.9 in older people's wards.

**Table 1:** Demographics of sample by quartile of adherence to scheduled vital signs observation intervals at night

	Lower quartile (*n* = 3)	Low‐mid quartile (*n* = 6)	Mid‐high quartile (*n* = 2)	Upper quartile (*n* = 6)	Total (*n* = 17)
Role					
Registered nurses	3	3	1	6	13
Student nurse/support worker	0	2	0	0	2
Support workers	0	1	1	0	2
Years of ward experience					
0–4 years	1	0	1	1	3
5–9 years	0	4	0	1	5
10–14 years	1	1	0	1	3
15–19 years	0	0	0	0	0
20–24 years	0	1	1	0	2
25–29 years	0	0	0	2	2
30+ years	1	0	0	1	2
Specialty					
Medical	1	0	0	0	1
Stroke Rehab	1	1	0	0	2
Older people (acute)	1	0	0	0	1
Oncology	0	3	0	0	3
Trauma & Orthopaedics	0	0	1	1	2
Emergency Medicine	0	0	0	2	2
Surgical	0	2	1	2	5
Gynaecology	0	0	0	1	1

Interviews, lasting between 19 and 61 min, were conducted face‐to‐face in a private room or over the telephone. The interview topic guide covered:

- patient characteristics, care needs and ward specialty,
- role responsibilities,
- views of the ward's protocol compliance
- barriers to complying with the protocol
- impact of other ward routines,
- attitude to complying with the protocol at night
- ward consequences following protocol non‐compliance
- views of protocol requirements
- impact (if any) of ward performance targets aimed at increasing compliance with the protocol

The lead author interviewed all participants. Data saturation was reached on the sixteenth interview, with no new insights gained in the final two interviews. Audio recordings were made with permission, stored securely and deleted after anonymized transcripts were created. Interviewees were given information sheets about the study including assurance their decision would not impact their role or promotion prospects. All interviewees provided written informed consent. Ward names have been anonymized and the personal details of interviewees removed from all reports. Data were stored in adherence with the Data Protection Act (1998).

Qualitative analysis was carried out using the constant comparative method described by Lofland, Snow, Anderson, and Lofland (2006). This is informed by grounded theory but permits the use of relevant top‐down codes. NVivo software was used. The lead author and fourth author each coded three transcripts, compared them, and reached a consensus on transcript coding and initial code framework. The remaining transcripts were divided between lead author and fourth author. Codes and coding were discussed through NVivo memos and regular meetings until consensus was reached.

## 4 FINDINGS

**Figure 2:**
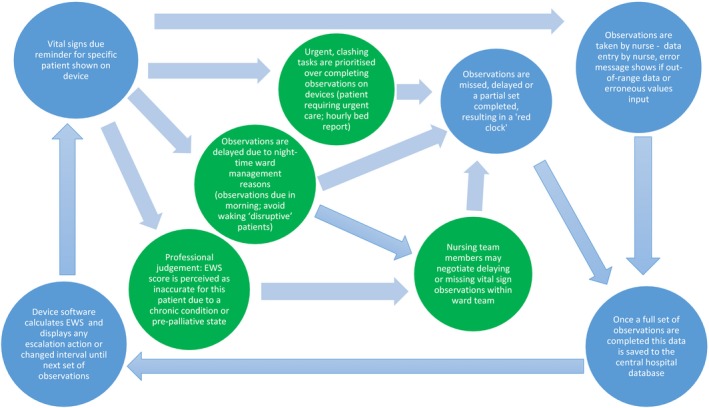
Nurse actions in relationship to use of devices and local ward expectation [Colour figure can be viewed at https://www.wileyonlinelibrary.com]

**Figure 3:**
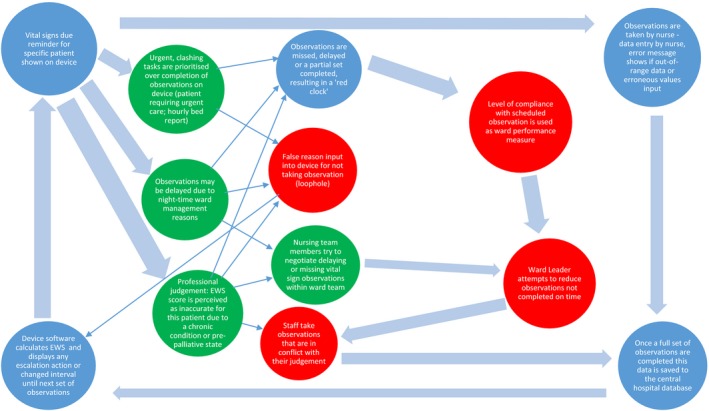
Impact of ward performance management by compliance with electronic early warning score protocol [Colour figure can be viewed at https://www.wileyonlinelibrary.com]

The first section describes nursing practices in relation to the protocol before performance targets were implemented. These are added to the process shown in Figure 1, with additional processes added in new circles (Figure 2). The final section explores how these processes changed when ward performance targets were implemented, with further circles added to Figure 3.

### 4.1 The role of an electronic EWS protocol before ward performance management

Interviewees described competing demands, which could interfere with their ability to take scheduled observations when the clock icon indicated they were due. This included clashes with other scheduled tasks such as completing the hospital‐wide bed occupancy database with patient information.

It has to be done hourly. We have an egg‐timer on the ward just to remind people to do it. So if your bell goes off in the middle of a set of obs, what are you going to do? You're going to be named and shamed the next day because you haven't managed to do it […] (RN9, Medium Compliance ward).

Interviewees also described missing scheduled observations to respond to rapid patient deterioration (crashes).

our main priority would be […] to see to [patient] crashes first [RN8, medium compliance ward]

Patients with chronic conditions such as chronic obstructive pulmonary disease (COPD), asthma and high blood pressure had chronically abnormal vital sign values that contributed to an elevated EWS value. This created an observation schedule perceived by many interviewees as inappropriately frequent. Interviewees described sometimes partially completing a vital signs set for these patient groups that would be recorded as a late or missed observation on the centralized EWS database and local devices (see Figure 2). They also described omitting vital sign measurements entirely for such patients (particularly at night), negotiating this at ward level and recording it in patient notes.

people that have COPD are on oxygen […] [which] scores a two [on the EWS] […] people with COPD don't really have sats above ninety‐two, that scores a two […] and their resps are high that scores a one, and if their pulse is tachy […]that scores another one, so that's already a seven which is an hourly obs. […] In that situation we always get the doctor to document that they do not require obs every hour (RN7, medium compliance ward)

Medical “outliers” (patients moved to a different specialty area to create bed space on another ward) could also have their vital signs missed at night. As shown below, this could even happen on wards where there was a high level of compliance with the protocol. This suggests that even when overall ward compliance is high, certain groups may be disproportionately affected by non‐compliance.

the medical outliers come up as [late], because technically they're fit enough to go home, so you know we wouldn't keep waking them up in the middle of the night to do them. (RN10, high compliance ward).

At night‐time, observations of people with dementia could be delayed or missed for non‐clinical reasons. Six interviewees told us people with dementia were not woken at night for scheduled observations because they were concerned about facing challenging behaviour, did not want to wake someone with trouble falling asleep, or their agitation on being woken might disrupt other patients’ sleep.

if somebody […] has severe end‐stage dementia, who can be quite aggressive and they've actually settled, are you going to go and do that person's blood pressure just because there’s a red clock? No, you're not. (RN8, medium compliance ward)

However, on some wards, interviewees claimed to have developed ways of taking vital sign observations at night that caused minimal distress to people with dementia.

so if we cannot do the upper, so we'll just do it at the bottom, at the lower torso. So for example, the saturation reading, you need to do it, so if you cannot do it in the fingers, we'll just do it in the toe. (SW4, high compliance ward)

Prior to the introduction of ward performance measures, reminders on the device to take a full set of vital sign observations were treated as advisory, used alongside the nurse's clinical judgement, ward management considerations and competing priorities. One interviewee said she had never woken a patient at night, another that a nurse‐in‐charge (on a different ward) had asked her not to take observations at night. However, some interviewees described benefits of having external reminders via the device:

time does fly when you're working in such a busy environment ‐ and it does bring your attention to, 'There's a red clock there; when was the last time this was done?’ (RN8, medium compliance ward)

Similarly, some nurses used the EWS to explore the reasons why a patient was unwell, using clinical judgement to decide on the next step, rather than relying on the EWS to make that decision.

Figure 2 shows how exceptions were negotiated before electronic data were used in ward performance management. This complements Figure 1 by highlighting the “hidden” work within a ward that is not recorded on the device (shown in additional circles) and is therefore absent from the protocol database.

### 4.2 Impact of ward performance measures using compliance data

Ten of the 17 interviewees described benefits associated with the introduction of ward performance targets. These included reducing the chance of accidentally overlooking patient monitoring, reducing the time required to delegate observations, redistributing observations throughout the shift, encouraging reconsideration of patient condition and increased patient contact time.

It was usually a case of handing over which obs you’d done and which obs you hadn't, which is definitely not as effective […]You've always got that risk that […] they would miss someone, they would forget to tell you […] and […] you saw [a patient] hadn't had obs for over 18 hours (RN7, medium compliance ward)

Significantly, although interviewees described the intervals required between observations as too short, they also acknowledged instances where unexpected deterioration had been detected more quickly.

you do sometimes catch ‐ when you think a patient's quite stable, and you actually do find that overnight they become unstable, that it is picked up. (RN11, medium compliance ward)

Interviewees reported pressure from ward managers to maintain and improve ward performance, meaning some interviewees would carry out observations even when this conflicted with their clinical judgement and was unpopular with patients.

Due to the inability to report reasons for omissions, interviewees described feeling penalized when prioritizing care of rapidly deteriorating patients.

it's a little bit demoralising […] because you suddenly feel, “Goodness, we're going to have not a good report […]” but actually you've been tied up in […] preserving life (RN4, Low Compliance ward)

The inability to report reasons for exceptions meant some staff used a loophole to avoid having an omission recorded.

I think some people do still find it frustrating that there isn't a way to turn it off when they know their patient's stable, but the only way you can do it is by [selecting a specific setting] which isn't ideal, but it stops the red clock and that's the way some people get around it (RN11, Medium Compliance ward)

This highlighted the existence of invisible non‐compliance – a disparity between the reality of nursing practice and the recorded electronic data.

Figure 3 summarizes these findings to show the impact of ward performance targets on staff protocol compliance. New actions are included in additional circles. The reasons why staff members judge that observations should be missed remain, but staff response differs due to increased pressure to demonstrate compliance with the protocol. The lack of a way to feedback reasons for non‐compliance led to nursing staff taking observations at odds with their clinical judgement or the use of a loophole to avoid penalization when care was omitted. This has the potential to erode a sense of professional autonomy or introduce new, unmonitored risks to patient safety.

## 5 DISCUSSION

This study highlighted some important issues relating to the wider use of electronic data in performance management of nursing compliance with protocols. Before this initiative began staff were able to negotiate exceptions with colleagues. However, once compliance targets were implemented this created covert behaviours that were invisible to the protocol database or forced protocol‐compliant care to be taken regardless of clinical (or ward management) judgement. This threatened nurses’ sense of autonomy and their perceived ability to provide personalized care to some patients. This reflects prior research that highlights the need to use clinical judgment to assess deterioration alongside EWS protocols and a continued discussion as to whether the development and use of such judgment is undermined by the use of EWS (Downey, Tahir, Randell, Brown, & Jayne, 2017).

The key limitation of this paper is its reliance on the accounts of nursing staff about their experience of using a protocol embedded within bedside handhelds. However, as can be seen from the quotations, nurses disclosed specific instances of ignoring the protocol. A greater understanding of the reasons for protocol non‐compliance could be gained through taking ethnographic observations of how nurses interact with the devices at the bedside.

This study allowed us to explore the role of professional judgement in the context of resistance to electronic protocols. As Allen has argued, protocols and supporting technologies often fail to account for the “invisible” work of nurses, which can involve emergent organizational care, focused around patients’ care trajectories. These can be complex and require protocol adaptation (Allen, 2014, 2019). Some of these concerns were supported to varying extents by the evidence base. One issue – also found in an evaluation of an electronic EWS system – was the lack of flexibility or feedback opportunities (Lang, Pinchin, Brown, & Sharples, 2016). In particular, the frequency of observations required for patients with chronic conditions (particularly COPD), where higher EWS scores reflected a chronic vital signs derangement, a difficulty highlighted by Wood, Chaboyer, and Carr (2019) in a recent review. Indeed, some research has used different thresholds to differentiate patients with respiratory conditions (CREW: Lobo, Lynch, & Casserly, 2015), while others suggest developing different algorithms for groups with distinct physiological profiles (Downey et al., 2017). However, an increasing body of research has found EWS (particularly NEWS) may be suitable for diverse patient groups, including those with COPD (Hodgson, Congleton, Venn, Forni, & Roderick, 2018; Hydes et al., 2018; Kovacs et al., 2016; Pimentel et al., 2018; Redfern et al., 2018). Interviewees also disputed the necessity of the frequency of observations overall and whether there was always a need to take a whole set of observations, which reflects gaps in the evidence base (Smith et al., 2017). Nevertheless, evidence identifying an elevated risk of death within 24 hr in patients with high EWS scores is strong (Prytherch, Smith, Schmidt, & Featherstone, 2010). Nurses’ treatment of the protocol as advisory reflects other research where protocols were treated as prescriptive guides, to be particularized for specific patients (Rycroft‐Malone, Fontenla, Seers, & Bick, 2009) or “circumvented, tinkered with and interpreted” (Berg, 1997).

However, as data from an emerging literature have demonstrated, nursing staff do not only use clinical judgement but also ward management decisions at night (Hope et al., 2018. Ward performance management using electronic data to assess compliance aims to deal with these kinds of nursing omissions. However, these findings demonstrate the use of loopholes to avoid detection and penalization, echoing the “passive resistance” used against the mandatory implementation of electronic patient records (Timmons, 2003), or the overt and covert resistance to algorithm‐adherence performance targets for 111 nurse advisors (Pope et al., 2017; Prichard et al., 2014; Ruston, 2006). In addition, these findings suggest that allowed margins of “non‐compliance” can drive systematic under‐monitoring of some groups under the hospital's radar. As described here, tightening compliance expectations risks reducing the visibility of missed care and creating new, undetected risks to patient safety.

Broad lessons can be learned from this study to increase patient safety and staff morale where electronic data are used in performance management of nursing compliance with protocols. Firstly, providing alternatives to replace the previously hidden mechanism of negotiating exceptions with colleagues, an example of an “invisible” example of nursing practice that may not be accounted for when new technology is introduced (Allen, 2014). This could be either a formal modification of the electronic protocol parameters, such as providing latitude around EWS baseline interval frequency (Jones et al., 2011). Such approaches reduce the need to use covert methods and generate evidence to fine‐tune protocols. Secondly, regularly monitoring data to explore whether some groups are more likely to receive less protocol‐based care. Finally, there should be a way to positively identify cohorts of patients where the protocol may be less appropriate. For these groups, valid alternative protocols with proportionate responses should be developed.

## 6 CONCLUSION

Standardization has the ability to reduce human error, and the use of digital data can uncover missed or suboptimal care. However, as this study has shown, it is important to approach the imposition of digital surveillance targets critically, and to understand reasons for passive resistance. This can help to avoid the use of covert strategies, which can undermine the purpose of using digital data in performance management by creating false readings.

## 7 INFORMATIVE

Electronic data are increasingly used to monitor nursing quality but little is known about the impact on nurses’ practice in busy hospital wards. This paper presents a case study of the use of information management and technology in nursing performance management: the use of electronic data to measure ward compliance with an early warning score protocol delivered by a handheld device. Interviews with 17 members of nursing staff found that introducing automated electronic systems to support nursing tasks decreases nursing burden but removes invisible mechanisms for negotiation that provide a balance between nursing judgement and standardized protocols. This can result in covert resistance that decreases patient safety.

## CONFLICT OF INTERESTS

At the time of the research, Portsmouth Hospitals NHS Trust (PHT) had a royalty agreement with The Learning Clinic (TLC) to pay for the use of PHT intellectual property within TLC’s VitalPAC product. VitalPAC is the system used to collect vital sign data in the current research. Until October 2015, PS and the wife of GBS were minority shareholders in The Learning Clinic. GBS worked for PHT from 1985 to 2011. PS is currently employed by PHT. Until 2015, GBS and PS were unpaid research advisers to TLC. JH, AR‐S, and PG have no conflict of interests.

## ETHICAL APPROVAL

The project received University of Southampton ethical approval (ERGO ref 10,813) on 30/03/2015 with governance approval gained from the Portsmouth Hospital Trust's Research and Development Office on 15/06/2015.
